# Optimization of Device-Free Localization with Springback Dual Models: A Synthetic and Analytical Framework

**DOI:** 10.3390/s25185696

**Published:** 2025-09-12

**Authors:** Jinan Li, Benying Tan, Yang Qin, Yaoyao Mo

**Affiliations:** School of Artificial Intelligence, Guilin University of Electronic Technology, Guilin 541004, China; ljn@mails.guet.edu.cn (J.L.); qinyang@guet.edu.cn (Y.Q.)

**Keywords:** device-free localization, sparse representation, transform learning, Springback penalty, difference of convex functions algorithm

## Abstract

In complex environments, traditional device-free localization (DFL) methods based on received signal strength (RSS) encounter difficulties in simultaneously achieving high accuracy and efficiency due to multipath effects and noise interference. These methods typically depend on convex sparsity regularization, which, despite its computational convenience, is insufficient in capturing the sparsity of signals. In contrast, non-convex sparsity regularization methods, while theoretically more capable of approximating ideal sparsity, are associated with higher computational complexity and a greater likelihood of getting stuck in local optima. To address these issues, this study proposes a synthetic model based on a novel weakly convex penalty function called Springback. This model combines a compression term ($\ell_{1}$) that promotes sparsity and a rebound term ($\ell_{2}$) that preserves signal amplitude, adjusting parameters to balance sparsity and computational complexity. Furthermore, to tackle the low efficiency of traditional synthetic models when dealing with large-scale data, we introduce a Springback-transform model based on an analytical transform learning framework. This model can directly extract sparse features from signals, avoiding the complex computational processes inherent in traditional synthetic models. Both models are solved using a difference of convex algorithm (DCA), significantly improving positioning accuracy and computational efficiency. Experimental results demonstrate that the proposed models exhibit high accuracy, low positioning error, and a short computation time across various environments, outperforming other state-of-the-art models. These achievements offer a new solution to the problem of DFL in complex environments, with high practical value and application prospects.

## 1. Introduction

Device-free localization (DFL) is an emerging wireless positioning technology [1,2,3] that can sense the location and movement of a target without the need for the target to carry any electronic devices. By detecting changes in wireless signals, DFL leverages its unique advantages of being device-free, cost-effective, and capable of high-precision positioning. These attributes make it highly promising for a wide range of applications, including smart home automation [4], emergency rescue [5], and security surveillance [6,7,8].

Early research focused primarily on localization using basic signal features such as received signal strength (RSS) [9,10] and time difference of arrival (TDOA) [11,12]. RSS, which is easy to obtain and cost-effective, has become a mainstream approach for localization in certain specific and constrained environments. By measuring the strength attenuation of multiple signal sources around the target and combining it with a path loss model to estimate distances, trilateration can be performed to determine the location. However, in complex indoor environments, multipath effects [13], dynamic noise interference, and non-line-of-sight (NLOS) signal propagation [14] make it difficult for models to balance the relationship between bias and variance, often falling into local optimal solutions, affecting the accuracy and stability of positioning.

To improve positioning accuracy and reliability, researchers in the field of DFL have begun to explore new methods to enhance the accuracy and efficiency of positioning. One important direction is the introduction of constrained optimization concepts into the training process of positioning models to optimize the performance of sparse representations, thereby significantly improving the accuracy and stability of positioning results. Although the traditional $\ell_{1}$ norm [15] and $\ell_{2}$ norm [16] help maintain the convexity of the problem and enable optimization processes that can identify the global minimum, they have limitations. The $\ell_{1}$ norm penalty tends to produce overly sparse solutions and is prone to underestimating the high-amplitude components of the solution, as it applies the same penalty to all components. Here, high-amplitude components refer to the elements in the model parameters or solution vector with larger values. These components typically represent the most important features or parameters within the model and significantly influence the model’s predictive capability. Consequently, although the $\ell_{1}$ norm penalty aids in feature selection, it may sacrifice some vital information, thereby affecting the model’s overall performance. In contrast, the $\ell_{2}$ norm penalty is sensitive to outliers and may affect the stability of the model when dealing with highly correlated features.

In comparison, the non-convex penalty constraint of smoothly clipped absolute deviation (SCAD) proposed by Fan et al. [17] and the $\ell_{p}$ norm proposed by Chartrand et al. [18] can theoretically approximate the ideal sparsity more closely. These methods impose more substantial penalties on small coefficients while keeping the penalties on large coefficients constant or even reducing them, thereby minimizing the bias in amplitude estimation and better handling complex scenarios. However, the optimization process for these non-convex methods is complex, with high computational costs, and they are prone to falling into local optima. Moreover, in existing DFL models, most adopt synthetic models. When dealing with large-scale data, these synthetic models face challenges such as low computational efficiency and inability to fully capture key signal features. Therefore, how to balance accuracy and efficiency in sparse modeling for DFL, avoid falling into local optima, and enhance the overall performance of the algorithm have become core challenges in the design of DFL.

Based on the above issues, this study proposes an independent and interpretable Springback model based on the weakly convex penalty function Springback penalty [19], as well as an independent and interpretable Springback model based on transform learning (IISpringback-transform). The core idea is to integrate two classic regularization strategies, a compressive term that promotes sparsity and a rebound term that protects signal amplitudes, thereby achieving a dynamically balanced elastic constraint. Here, signals refer to the raw information or features extracted from the data, which are crucial for the model’s predictive or estimation tasks. In positioning applications, these signals may be related to RSS or other relevant measurements, representing the position or state of objects in the environment. Specifically, based on the traditional $\ell_{1}$ norm, a negative $\ell_{2}$ norm is introduced as a correction term, with the weights of the two terms adjusted by a parameter θ. When the signal components are small, the $\ell_{1}$ term dominates to enhance sparsity; however, when the signal amplitude is large, the negative $\ell_{2}$ term generates a counteracting correction force, effectively alleviating the issue of underestimation of the main signal amplitude caused by excessive compression in traditional methods. This design has been mathematically proven to exhibit "weak convexity", retaining the ability of non-convex methods to flexibly approximate ideal sparsity while avoiding the pitfalls of falling into local optima that are common in fully non-convex optimization, thus laying the foundation for an efficient solution. Furthermore, the IISpringback-transform model leverages the benefits of transform learning [20,21,22]. It is an analytical model that analyzes signal data directly by learning an analytical transformation. This approach allows the transformation to process the signals more directly.

In light of the weakly convex optimization characteristics of the Springback penalty, this study employs the difference of convex functions algorithm (DCA) [23,24] for an efficient solution. This algorithm decomposes the complex non-convex problem into the difference of two convex functions, approximating the global optimal solution through a non-convex proximal splitting algorithm. In each iteration, the algorithm only needs to solve a standard convex optimization subproblem, significantly reducing computational complexity compared to directly handling the non-convex model. Through this method, this study achieves a dual improvement in accuracy and efficiency in the field of device-free localization, offering a new approach and methodology for localization problems in complex environments.

The main contributions of this study are as follows:

- This study innovatively proposes two DFL models based on the Springback penalty—namely, IISpringback and IISpringback-transform. The Springback constraint effectively addresses the challenges of accuracy and efficiency in RSS-based DFL under complex environments by integrating $\ell_{1}$ convex penalties with $\ell_{2}$ convex penalties, significantly enhancing the positioning performance. Furthermore, to tackle the weakly convex optimization issue associated with the Springback penalty, we employed the DCA and the non-convex proximal splitting algorithm to achieve efficient solutions, further enhancing the practicality and efficiency of the models.
- The IISpringback-transform model adopts an analytical transform learning method, which avoids the complex calculation of matrix decomposition in traditional synthetic models, improves calculation efficiency, and maintains high-precision localization capabilities in complex environments, providing an efficient and accurate solution to the problem of DFL.
- This study has been extensively experimentally verified on indoor and outdoor datasets. The experimental results show that the proposed model exhibits excellent performance under different environmental conditions, further proving its effectiveness and universality in device-free localization tasks in complex environments.

The structure of this paper is as follows. The second part is an introduction to related work. The third part describes the details of our model. The fourth part is the experimental part. The last part is our summary and outlook.

## 2. Related Works

Youssef et al. [25] first introduced the concept of a device-free passive (DfP) system, which is capable of detecting, tracking, and identifying target entities that do not carry any devices and do not actively participate in the localization process in wireless environments. This laid the foundation for the subsequent development of DFL technologies. Wilson et al. [26] proposed an innovative radio tomographic imaging (RTI) technique that localizes and images physical objects by monitoring the changes in RSS within wireless networks, thereby providing a new approach for device-free detection in indoor environments. Zhang et al. [27] presented a device-free localization system named RASS, which is based on RSS and can track mobile objects that do not carry any communication devices in real time and with high accuracy within the coverage area of wireless sensor networks. Subsequently, Zhang et al. [28] proposed another system based on Received Signal Strength Indicator (RSSI). By constructing a signal dynamic model, this system enables the tracking of objects without transmitters or receivers in environments covered by wireless sensor networks.

However, RSS is highly susceptible to multipath effects and dynamic environmental changes, leading to a contradiction between the static nature of the fingerprint database and signal fluctuations. Theoretically, calculating the target position by measuring the TDOA of signals at different receivers can overcome the distance estimation limitations of RSS. However, this method requires extremely high synchronization accuracy, and the time difference errors caused by NLOS propagation are difficult to compensate for.

To address the challenges posed by complex environments, research has gradually shifted towards wireless localization algorithms based on sparse representation and dictionary learning. Wang et al. [29] proposed a device-free localization method based on compressive sensing. By employing a dynamic statistical model and the Bayesian greedy matching pursuit (BGMP) algorithm, this method significantly improved localization accuracy and tracking performance in wireless network environments. Huang et al. [30] introduced a device-free localization method based on sparse coding. Using the sparse coding iterative shrinkage thresholding algorithm (SC-ISTA) and subspace techniques, this method significantly enhanced the accuracy and efficiency of device-free object localization in wireless sensor networks in high-dimensional data environments. Zhang et al. [31] proposed a method that combines non-convex regularization (Generalized Minimax Concave, GMC) with an adaptive relaxation localization (ARL) criterion to improve localization accuracy. By employing the forward–backward splitting (FBS) algorithm to identify the global optimal solution, this method achieved accurate and robust localization performance in challenging environments. Jiang et al. [32] proposed a device-free indoor localization method that integrates kernel transformation and dictionary learning in high-dimensional spaces. By optimizing localization accuracy through the kernel k-singular value decomposition (KKSVD) algorithm, this method addressed the linear inseparability problem of low-dimensional localization data. Xia et al. [33] proposed a device-free localization method that incorporates independent interpretable sparse coding and minimax concave penalty (MCP) regularization. By solving the non-convex optimization problem using the DCA, this method significantly improved localization accuracy and efficiency.

In existing sparse representation-based DFL methods, the sparse constraints are either convex functions or non-convex functions. For instance, the Elastic Net, proposed by Zou et al. [34], is a strictly convex model that combines the $\ell_{1}$ and $\ell_{2}$ norms to address the issue of feature correlation. This method performs well in handling high-dimensional data and feature selection, reliably finding the global optimal solution without falling into local optima. However, in complex scenarios where features have highly nonlinear relationships, its flexibility may be limited compared to non-convex optimization methods.

On the other hand, non-convex functions are also used as sparse constraints, but these non-convex methods have complex optimization processes, high computational costs, and a propensity to get stuck in local optima. Moreover, most existing device-free localization models employ synthetic models. These synthetic models face challenges such as low computational efficiency and an inability to fully capture key signal features when dealing with large-scale data. Therefore, balancing accuracy and efficiency in sparse modeling for device-free localization, avoiding falling into local optima, and enhancing the algorithm’s overall performance have become core challenges in the design of DFL algorithms.

## 3. Model and Formulation

**Figure 1:**
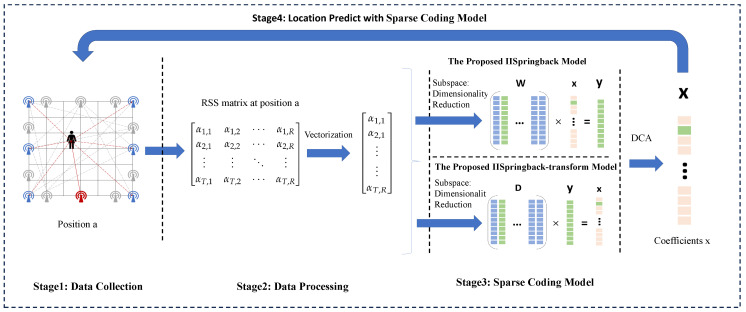
Illustrations of data patterns and flowcharts for the two proposed models.

In this section, we delve into the process of RSS data collection and processing and elaborate on the details of the two DFL models proposed based on the Springback penalty function. The data model diagram and flowchart of the two proposed models are shown in Figure 1, and the main notations used in this paper are summarized in Table 1.

### 3.1. Data Collection and Processing of RSS

#### 3.1.1. Stage of Dictionary Construction

**Figure 2:**
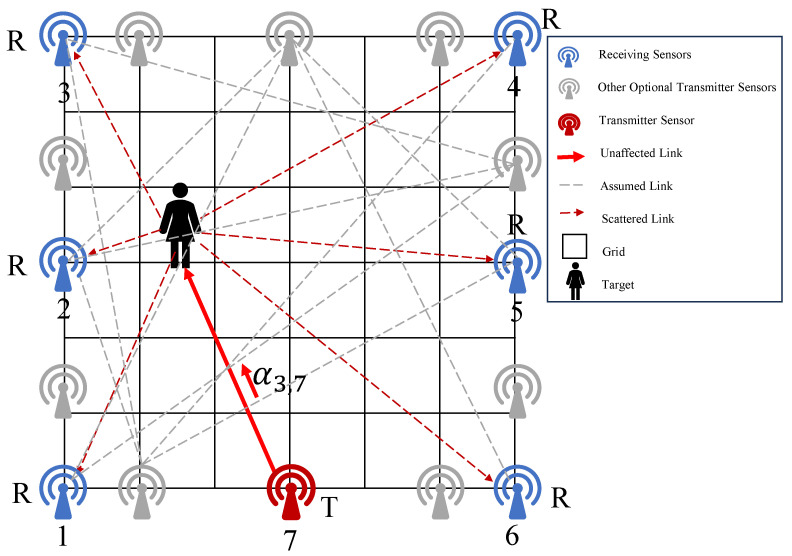
Schematic diagram of the device-free localization system model.

As shown in Figure 2, in a DFL system, the monitoring area can be discretized into a grid of cells. The target in the system will affect the RSS measurement at the anchor point (in a DFL system, wireless sensor nodes are typically referred to as anchor points, and wireless sensors encompass both transmitting and receiving sensors). When the target moves between different grids, the RSS measurement value changes accordingly, thereby forming different joint signal configurations.

The received RSS is measured in milliwatts (mW) and can be normalized to decibel-milliwatts (dBm), which represents the absolute value of the received signal strength. The unit dBm is used to denote the power level. When the receiver successfully captures all wireless signals transmitted by the transmitters, the dBm value of the wireless signal is defined as 0; hence, the converted values are typically negative. To facilitate the computation in sparse coding and dictionary learning algorithms, the unit was not converted to dBm in this experiment.

In the DFL system, assume that there are *T* transmitting sensors and *R* receiving sensors. Define $\alpha_{i,j}$ as the RSS measurement value received by the *i*-th receiving sensor from the *j*-th transmitting sensor, where i∈{1,2,…,R} and j∈{1,2,…,T}. When the DFL system is running, a matrix containing the RSS measurement values of all links can be constructed in the following form:

(1) $$\begin{array}{cc} \; & \gamma ={\left[\alpha_{1},\alpha_{2},\ldots ,\alpha_{R}\right]}^{T}\\ \; & =\left[\begin{array}{cccc} \alpha_{1,1} & \alpha_{1,2} & \ldots & \alpha_{1,T}\\ \alpha_{2,1} & \alpha_{2,2} & \ldots & \alpha_{2,T}\\ \vdots & \vdots & \ddots & \vdots \\ \alpha_{R,1} & \alpha_{R,2} & \ldots & \alpha_{R,T} \end{array}\right]\in R^{R\times T} \end{array}$$

In this monitoring system, it is assumed that the monitoring area is discretized into a grid consisting of M cells, and each grid can be used as a reference point (RP); that is, there are *M* RP points in total, corresponding to *M* categories. Take the *n*th RP point as an example, where n≤M. Specifically, the target is placed at the *n*th RP point, and *z* experiments are performed to obtain *z* groups of RSS matrices, $\left\{\gamma_{n1},\gamma_{n2},\cdots ,\gamma_{nb},\cdots ,\gamma_{nz}\right\}$. Define $\gamma_{n,b}$ as the *b*th RSS matrix measured at the *n*th reference point, where b∈{1,2,…,z}.

Subsequently, each RSS matrix is vectorized to obtain the corresponding vectorized vector.

(2) $$w_{nb}=vec\left(\gamma_{nb}\right)\left(1\leq b\leq z\right)$$

Here, $\gamma_{nb}$ is the *b*-th sample vector of the *n*-th RP. Then, we arrange the *z* sample vectors of the *n*-th RP into columns of the matrix $W_{n}$, which is constructed as follows:

(3) $$\begin{array}{cc} W_{n}=\left[w_{n1},w_{n2},\ldots ,w_{nb},\ldots ,w_{nz}\right]\in & R^{(R\times T)\times z},\\ \; & \left(1\leq n\leq K\right) \end{array}$$

After completing the traversal of all positions, we integrate the matrices of these positions to construct a comprehensive perception matrix (dictionary):

(4) $$\begin{array}{cc} W & =\left[W_{1},W_{2},\ldots ,W_{n},\ldots W_{M}\right]\in R^{\left(R\times T)\times (M\times z\right)} \end{array}$$

Simultaneously, the labels are concatenated as follows: $\mathrm{lab}=\left\{1,1,\ldots ,m,m,\ldots ,M\right\}\in R^{M\times z}$. Each label is repeated *z* times, matching the order of samples in the dictionary. Each unique location label corresponds to a specific two-dimensional coordinate, with each sample having an associated label. The labels of the test signals are used to assess the accuracy of the predicted locations during testing.

#### 3.1.2. Construction of the Test Signal

We assume that when the test target is placed at the *c*-th RP position in the DFL, an observation signal y will be generated. This signal can be approximated by the *c*-th sample matrix $W_{c}$, as described below:

(5) $$\begin{array}{cc} y & =W_{c}x_{c}\\ \; & =\Sigma_{l=1}^{z}w_{cl}x_{cl}\\ \; & =x_{c1}w_{c1}+x_{c2}w_{c2}+\ldots +x_{cz}w_{cz},\left(1\leq c\leq M\right)\; \end{array}$$

Here, $x_{c}={\left[x_{c1},x_{c2},\cdots ,x_{cz}\right]}^{T}\in R^{z}$ is a vector consisting of sparse coefficients, *z* represents the number of experimental trials, and $x_{cj}\in R$ is the coefficient of each item.

This representation is chosen because when there is a high degree of correlation between signals, the corresponding coefficients also exhibit correlation. Since most columns are correlated, the terms on the right side of the equation typically cannot remain independent during the construction of the RSS dictionary. By adding independently interpretable regularization, we can precisely set the coefficients $x_{cl}$ of certain correlated variables to zero. This process not only ensures the independence among the remaining active variables but also prevents redundancy in the representation of y. In this way, each coefficient accurately reflects the contribution of its corresponding independent variable, thereby significantly enhancing the model’s interpretability.

#### 3.1.3. Model Construction

The DFL problem is essentially a “sparse representation-based classification problem”. The location of the target can be represented as a sparse linear combination of atoms (the column vectors that make up the basis of the sparse representation dictionary). In other words, if a target is located in or moves through a specific spatial region, the wireless signal associated with that region will change, and these changes can be used to construct a sparse vector in which only the elements corresponding to the actual region where the target is located are non-zero, while the other elements are zero. By solving this sparse representation problem, the DFL system can determine the target’s exact location.

However, this equation is often an underdetermined linear system, and the solutions for *x* are infinite, so we need to add sparse constraints to find a specific solution. The resulting target equation is

(6) $$\min_{x}L\left(x\right)=\frac{1}{2}{∥y-Wx∥}_{2}^{2}+\lambda B_{1}\left(x\right)+\beta B_{2}\left(x\right)$$

The first term is the data fidelity term, where y is the observed noisy signal, W is the dictionary, and x is the sparse coefficient. This term measures the difference between the model prediction and the actual observation, and the goal is to make the prediction as close to the actual observation as possible. $B_{1}\left(x\right)$ is a regularization term used to introduce additional constraints or prior knowledge to improve the accuracy and robustness of localization. λ and β are both regularization parameters. $B_{1}\left(x\right)$ usually uses the $\ell_{1}$ or $\ell_{2}$ norm of a convex function or a non-convex function. In this article, we use the Springback constraint as a regularization constraint:

(7) $$R_{\theta }^{\mathrm{SPB}}\left(x\right):={∥x∥}_{1}-\frac{\theta }{2}{∥x∥}_{2}^{2}\left(\theta >0\right)$$

where, θ is the Springback penalty parameter, and in this study, θ is set to 0.7. The specific reasons for this choice can be found in detailed explanations within [19].

$B_{2}\left(x\right)$ is used to characterize the coherence between dictionary columns [33].

(8) $$B_{2}\left(x\right)=\frac{1}{2}{\left|x\right|}^{T}G\left|x\right|$$

Here, G is a symmetric matrix, where the component $G_{ij}\geq 0$ represents the coherence between dictionary columns $G_{i}$ and $G_{j}$. The coherence of a matrix is an important indicator to measure the correlation between its column vectors. When building a dictionary using highly correlated data, fully considering the coherence of the matrix is crucial to obtaining a unique sparse solution and achieving precise localization. G is defined as follows:

(9) $$G_{\mathrm{ij}}=\left\{\begin{array}{cc} \left|W_{i}^{T}W_{j}\right|, & i\neq j\\ 0, & i=j \end{array}\right.$$

#### 3.1.4. Solution of the Model

We need to solve (6) for the coefficient x:

(10) $$\begin{array}{cc} x & =\left\{x_{1},x_{2},\ldots ,x_{n}\right\}\\ \; & =\left\{x_{11},x_{12},\ldots ,x_{pj},\ldots ,x_{Mz}\right\}\\ \; & for\;p=1,\cdots ,M;\;j=1,\cdots ,z \end{array}$$

According to (10), we first calculate each $x_{p}$, which is obtained by summing $x_{pj}$, where j ranges from 1 to z. Then, we transform *x* into $x^{*}$ as follows:

(11) $$x^{*}=\left\{x_{1}^{*},\cdots ,x_{p}^{*},\cdots ,x_{M}^{*}\right\},\;for\;x^{*}\in R^{M\times 1}$$

During the solution process, if the solution vector *x* contains only a single non-zero element, then the RP position corresponding to this element can be considered as the specific position of the target. However, this is a rare case because usually, when the target position is predicted at the *s*-th grid, there will be multiple non-zero elements in the solution vector *x*. In this case, we can determine *s* as follows:

(12) $$s=argmax\left\{\begin{array}{c} x_{1}^{*},x_{2}^{*},...,,x_{p}^{*},...,x_{M}^{*} \end{array}\right\}$$

### 3.2. A Springback-Based Synthetic Independently Interpretable DFL Model

#### 3.2.1. Establishment of the Target Equation

We chose Springback as the sparsity constraint because it is a weakly convex function. The advantages of such weakly convex functions have been verified in various theoretical analyses and algorithm designs [35,36]. Its definition is as follows:

If the function $x\mapsto f\left(x\right)+\frac{\theta }{2}{∥x∥}_{2}^{2}$ is convex, then the function $f:R^{n}\to R$ is θ-weakly convex.

The Springback constraint can be regarded as a balance between the convex $\ell_{1}$ penalty and the non-convex penalty. The convex $\ell_{1}$ penalty has good processability in theoretical analysis and numerical calculation because convex optimization problems tend to have global optimal solutions. However, when capturing the sparsity of signals, it is likely to underestimate the high-amplitude components, resulting in unsatisfactory effects in achieving the sparsity target. On the other hand, the non-convex penalty formally approaches the $\ell_{0}$ norm, which measures sparsity, enabling it to better capture the sparse characteristics of signals. Nevertheless, in the solution of non-convex optimization problems, there are issues such as the absence of a global optimal solution and the existence of multiple local optimal solutions, which increases the complexity of the algorithm.

The Springback penalty integrates the advantages of both. It not only inherits the convenience of analysis and calculation of the $\ell_{1}$ penalty but also better approximates the $\ell_{0}$ penalty by introducing the term $-\frac{\theta }{2}{∥x∥}_{2}^{2}$, effectively balancing sparsity and computational complexity and providing a more efficient solution. In addition, in the function optimization of the “strongly convex + weakly convex” combination, the strongly convex function serves as the data fidelity term to ensure that the recovered signal is similar to the original data, while the Springback penalty acts as the sparse driving penalty term, reducing the non-zero estimation bias of the convex penalty term and thus improving the quality and accuracy of signal recovery.

The introduction of the coherence constraint can enhance the interpretability of the model. By excluding related variables, it enables each coefficient to represent the contribution of an independent variable, thus making the model decomposable and easier to understand. Under the condition of low coherence, each column vector can independently contribute to signal identification, which helps to accurately find the unique solution and improve the accuracy of sparse recovery. In addition, it can also assist the model in feature selection, screening out key features from highly correlated data, preventing the model from learning redundant information, thereby preventing overfitting, enhancing the generalization ability of the model, and improving the stability and reliability of the model across different datasets and scenarios.

Based on the content shown in (6)–(8), this research proposes a novel independent and interpretable DFL model based on Springback, IISpringback. The model incorporates sparse constraints with weak convex properties and coherence regularization as key components of the loss function. Specifically, the objective equation can be stated as follows:

(13) $$\min_{x}L\left(x\right)=\frac{1}{2}{∥y-Wx∥}_{2}^{2}+\lambda \left({∥x∥}_{1}-\frac{\theta }{2}{∥x∥}_{2}^{2}\right)+\frac{\beta }{2}{\left|x\right|}^{T}G\left|x\right|$$

where y is the observed signal, W is the dictionary, and x is the sparse coefficient. λ and β are the regularization parameters for the Springback constraint and the coherence constraint, respectively. θ is the model parameter for the Springback penalty, and G is a symmetric matrix, which is defined in (9).

#### 3.2.2. Updating Sparse Coefficients Based on the DCA

In this study, we directly update the solution x for (13) using the DCA. To meet the requirements of the DC algorithm, we can directly formulate the L(x) objective equation as a DC program because it takes the form of the difference between two convex functions.

(14) $$\kappa =\inf\{L\left(x\right):=h_{1}\left(x\right)-h_{2}\left(x\right)|x\in R^{M}\}$$

Here, we define DC components $h_{1}\left(x\right)$ and $h_{2}\left(x\right)$ corresponding to two lower semi-continuous proper convex functions, (15) and (16).

(15) $$\begin{array}{cc} \; & h_{1}\left(x\right)=\frac{1}{2}{∥y-Wx∥}_{2}^{2}+{\lambda ∥x∥}_{1}+\frac{\beta }{2}{\left|x\right|}^{T}G\left|x\right| \end{array}$$

(16) $$\begin{array}{c} h_{2}\left(x\right)=\frac{\lambda \theta }{2}{∥x∥}_{2}^{2} \end{array}$$

Since $h_{1}\left(x\right)$ and $h_{2}\left(x\right)$ are proper convex functions, their conjugate convex functions $h_{1}^{*}\left(\tilde{x}\right)$ and $h_{2}^{*}\left(\tilde{x}\right)$ are defined as follows:

(17) $$h_{1}^{*}\left(\tilde{x}\right)=\sup\left\{\langle \tilde{x},x\rangle -h_{1}\left(x\right)|x\in R^{M}\right\}$$

(18) $$h_{2}^{*}\left(\tilde{x}\right)=\sup\left\{\langle \tilde{x},x\rangle -h_{2}\left(x\right)|x\in R^{M}\right\}$$

Then, the dual problem of (14) can be formulated as follows:

(19) $$\kappa =\inf\{h_{1}^{*}\left(\tilde{x}\right)-h_{2}^{*}\left(\tilde{x}\right)|\tilde{x}\in R^{M}\}$$

Next, we need to construct two sequences $\left\{x^{\left(k\right)}\right\}$ and $\left\{{\tilde{x}}^{\left(k\right)}\right\}$ to alternately solve the primal problem (14) and the dual problem (19), because in this way we can utilize duality to approximate the optimal solution from different perspectives.

Step 1: with ${\tilde{x}}^{\left(k\right)}\in \partial h_{2}\left(x^{\left(k\right)}\right)$, solve (19):

(20) $$\partial h_{2}\left(x^{\left(k\right)}\right)=\lambda \theta x^{\left(k\right)}$$

Step 2: with $x^{k+1}\in \partial h_{1}^{*}\left(x^{\left(k\right)}\right)$, solve (14):

(21) $$\begin{array}{cc} \; & x^{k+1}\in \underset{x}{\mathrm{argmin}}\frac{1}{2}{∥y-Wx∥}_{2}^{2}+{\lambda ∥x∥}_{1}+\frac{\beta }{2}{\left|x\right|}^{\;T}G\left|x\right|-\langle x,{\tilde{x}}^{\left(k\right)}\rangle \end{array}$$

Here, $\partial h_{2}\left(x\right)$ represents the subdifferential of $h_{2}\left(x\right)$. In this model, when $h_{2}\left(x\right)$ is known and differentiable, we can analytically calculate the element of the subdifferential of $h_{2}\left(x\right)$, denoted as ${\tilde{x}}^{\left(k\right)}$, as follows:

(22) $${\tilde{x}}^{\left(k\right)}=\nabla h_{2}\left(x^{\left(k\right)}\right)$$

For (21), we apply the non-convex proximal splitting algorithm [37,38] and solve it through a loop iteration method:

(23) $$x^{k+1}=Prox_{\pi ,,h_{12}}\left(x^{k}-\frac{1}{\pi^{k}}\nabla T\left(\cdot \right)\right)$$

(24) $$T\left(\cdot \right)=\frac{1}{2}{∥y-Wx∥}_{2}^{2}-\langle x,{\tilde{x}}^{\left(k\right)}\rangle$$

According to (23) and (24), we will obtain the update rule for the sparse coefficients.

(25) $$x^{k+1}=Prox_{\pi ,h_{12}}\left(x^{k}+\frac{1}{\pi }\left(W^{T}\left(y-Wx^{k}\right)+{\tilde{x}}^{\left(k\right)}\right),m\right)$$

where m=λ+βG|x|, $Prox_{\pi ,h_{12}}$ represents the soft threshold operator for function $h_{12}\left(x\right)=\lambda {∥x∥}_{1}+\frac{\beta }{2}{\left|x\right|}^{T}G\left|x\right|$, which is defined as follows:

(26) $$Prox_{\pi ,h_{12}}\left(x,m\right)={\left(\beta G+I\right)}^{-1}sign\left(x\right)max\left\{|x|-m\right\}$$

Here, π is the step size, β is the regularization parameter for the correlation constraint, and sign(x) is the sign function.

The proposed algorithm is summarized in Algorithm 1.
**Algorithm 1** IISpringback.**Require:** signal y, dictionary W, positive regularization parameters λ, β, θ and π is greater than the largest eigenvalue of $W^{T}W$**Ensure:** $x=x^{k}$  1:Initialize $x^{0}=0,k=1$  2:**for** k=1 to maxiteration **do**  3:   ${\tilde{x}}^{k-1}\in \partial h_{2}\left(x^{k-1}\right)$ by (16) = $\lambda \theta x^{k-1}$ (20)  4:   $x^{k}\in \underset{x}{\mathrm{argmin}}\frac{1}{2}{∥y-Wx∥}_{2}^{2}+\lambda {∥x∥}_{1}+\frac{\beta }{2}{\left|x\right|}^{T}G\left|x\right|-\langle x,{\tilde{x}}^{k-1}\rangle$  5:   $m=\lambda +\beta G\left|x^{k-1}\right|$  6:   $x^{k}=Prox_{\pi ,h_{12}}\left(x^{k-1}+\frac{1}{\pi }\left(W^{T}\left(y-Wx^{k-1}\right)+{\tilde{x}}^{k-1}\right),m\right)$  7:**end for**  8:**if** *k* = maxiteration or $x^{k}$ has completed convergence **then**  9:   Establish the location by using (12).10:**end if**

### 3.3. The IISpringback-Transform Model Based on Analytical Transform Learning for DFL

#### 3.3.1. Review of Transform Learning (TL)

Transform learning (TL) [39,40] employs an analysis transform to directly analyze the signal. It processes the signal data directly by learning a specific transform, aiming to find a transformation method that can enable the transformed signal to better represent its essential characteristics, which are often sparse. The core of TL lies in the fact that the learned transform can map the signal to a new space where sparse signal features are more easily captured and utilized. The specific objective equations are as follows:

(27) $$\min_{x}{∥x-D\cdot y∥}_{F}^{2}+\lambda B_{1}\left(x\right)$$

where

- y represents the input signal;
- D represents the transform;
- x represents the coefficient;
- ${∥\cdot ∥}_{F}$ denotes the Frobenius norm;
- $B_{1}\left(x\right)$ denotes the sparsity constraint;
- λ is a regularization parameter.

This approach enables TL to process the signal more directly. TL uses an analysis transform to directly analyze the signal, and during the analysis process, each analysis transform contributes equally to the signal analysis. Additionally, TL uses an analysis transform regularizer to avoid trivial solutions, facilitating the discovery of more valuable signal features.

#### 3.3.2. Establishment of the Target Equation

Based on the above IISpringback algorithm, this study further explores the application of transform learning. Unlike the synthetic framework, transform learning adopts an analytical framework. In the analytical framework, the input signal is directly analyzed and processed instead of reconstructing the signal by synthesis. This analytical framework shows higher efficiency when processing large-scale datasets because it avoids the complex calculation process required for matrix decomposition of each signal sample in synthetic models.

Therefore, this study proposes a Springback model based on transform learning for DFL, called IISpringback-transform. The objective equation of the model is as follows:

(28) $$\min_{x}L\left(x\right)=\frac{1}{2}{∥x-Dy∥}_{2}^{2}+{\lambda (∥x∥}_{1}-\frac{\theta }{2}{∥x∥}_{2}^{2})+\frac{\beta }{2}{\left|x\right|}^{T}G\left|x\right|$$

where y is the observed signal, D is the transform matrix (dictionary), and x is the sparse coefficient. λ and β are the regularization parameters for the Springback constraint and the coherence constraint, respectively. θ is the model parameter for the Springback penalty, and G is a symmetric matrix, which is defined in (9).

#### 3.3.3. Updating Sparse Coefficients Based on the DCA

To solve the objective equation, we also use the DCA and the non-convex proximal splitting algorithm. This solution process is similar to the method for solving the Springback problem; the main difference is that (28) is adjusted accordingly. Specifically, we modify the equation as follows:

(29) $$x^{k+1}=Prox_{\pi ,h_{12}}\left(x^{k}+\frac{1}{\pi }\left(D^{T}y-x^{k}\right)+{\tilde{x}}^{\left(k\right)}\right),m)$$

The proposed algorithm is summarized in Algorithm 2.
**Algorithm 2** IISpringback-transform.**Require:** signal y, dictionary D, positive regularization parameters λ, β,θ and π is greater than the largest eigenvalue of $D^{T}D$**Ensure:** $x=x^{k}$  1:Initialize $x^{0}=0,k=1$  2:**for** k=1 to maxiteration **do**  3:   ${\tilde{x}}^{k-1}\in \partial h_{2}\left(x^{k-1}\right)$ by (16) = $\lambda \theta x^{k-1}$ (20)  4:   $x^{k}\in \underset{x}{\mathrm{argmin}}\frac{1}{2}{∥x-Dy∥}_{2}^{2}+\lambda {∥x∥}_{1}+\frac{\beta }{2}{\left|x\right|}^{T}G\left|x\right|-\langle x,{\tilde{x}}^{k-1}\rangle$  5:   $m=\lambda +\beta G\left|x^{k-1}\right|$  6:   $x^{k}=Prox_{\pi ,h_{12}}\left(x^{k-1}+\frac{1}{\pi }\left(D^{T}y-x^{k-1}\right)+{\tilde{x}}^{k-1}\right),m)$  7:**end for**  8:**if** *k* = maxiteration or $x^{k}$ has completed convergence **then**  9:   Establish the location by using (12).10:**end if**

## 4. Experiments and Results

In this section, we first introduce the datasets and evaluation metrics related to the experiments in Section 4.1 and Section 4.2, as well as an efficient dimensionality reduction method in Section 4.3. Then, in Section 4.4 and Section 4.5, we compare our model with several previously optimal sparse coding models under both indoor and outdoor conditions to comprehensively assess the model’s performance in different environments. Subsequently, in Section 4.6, we conduct two sets of ablation experiments in an indoor setting to demonstrate the impact of transform learning and dictionary column coherency on model performance. Finally, in Section 4.7, we provide an in-depth discussion and analysis of the experimental results.

All experiments were conducted in a standardized hardware and software environment. Specifically, the experiments were performed on a computer with a Windows 64-bit operating system installed with MATLAB 2024b. The computer was equipped with 16 GB of RAM and an Intel(R) Core(TM) i7-11800 CPU.

### 4.1. Dataset Overview

#### 4.1.1. Introduction to the Outdoor Public Experiment

For the outdoor environment, we utilized the outdoor experimental dataset provided by the SPAN (Sensing and Processing Across Networks) Laboratory at the University of Utah [26]. This dataset offers authentic outdoor environmental conditions for experiments, facilitating the verification of the model’s applicability and robustness in complex and variable outdoor scenarios. The specifics of this dataset are as follows.

**Figure 3:**
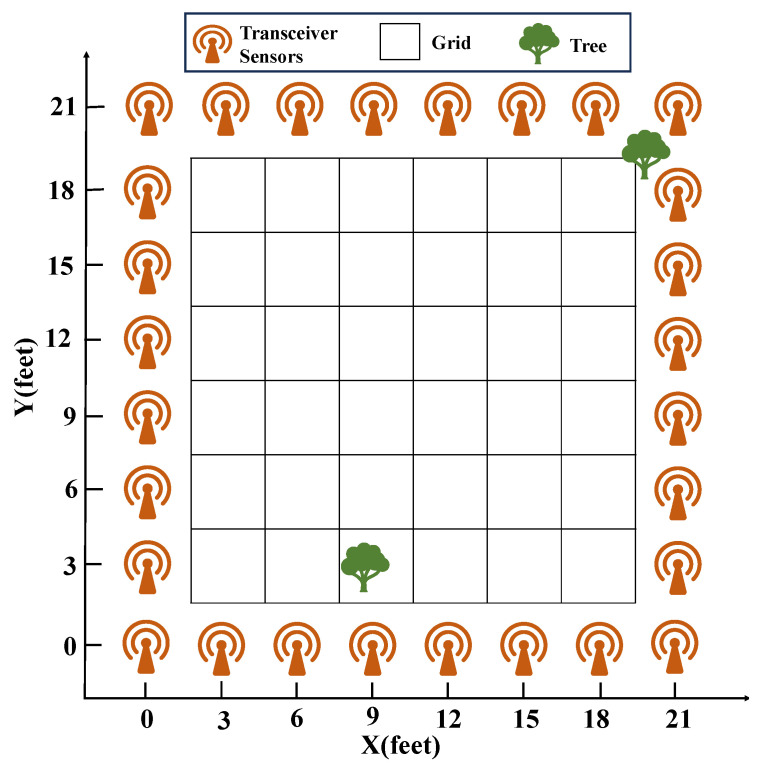
Schematic diagram of the DFL experimental setup designed by the SPAN Lab at the University of Utah.

As illustrated in Figure 3, the outdoor experiment was conducted within a 21-foot by 21-foot square area, surrounded by 28 wireless sensor nodes spaced evenly at 3-foot intervals. The entire network system was mounted on stands elevated three feet above the ground. Within the monitoring area, there were two trees with a circumference of approximately three feet. The target of the experiment was an individual with a height of 1.85 m. The wireless network was divided into 36 grids, 1 of which was occupied by trees; hence, the target could only be located within these 35 grids. The wireless network system was composed of TelosB wireless sensor nodes manufactured by Crossbow Technology. These nodes operate in the 2.4 GHz frequency band and communicate using the IEEE 802.15.4 standard. A base station node was set up to monitor all network traffic and transmit the collected data to a laptop via USB for further analysis.

In the experiment, the sensor network consisted of 28 sensors, each capable of both transmitting and receiving functions. However, at any given time, only 1 sensor acted as the transmitter while the remaining 27 sensors functioned as receivers. When the target was present at a fixed location within the sensor network (a total of 35 locations), the first sensor transmitted radio signals, and the other 27 sensors received these signals, with the transmission and reception process repeated 30 times. Subsequently, the next sensor would transmit signals, with all other sensors receiving the signals, until the last sensor had transmitted and the others had received the radio signals. All the saved information constituted the complete location information for the target at that position. The RSS information at each location was divided into two parts: 25 trials were used to construct a dictionary, and the remaining 5 trials served as test signals. It is important to note that the system was calibrated by taking RSS measurements when there was no target movement within the network.

Table 2 presents the values of key parameters for the two proposed models in the outdoor experimental environment.

#### 4.1.2. Introduction to the Indoor Experiment

For the indoor environment, we tested using a single-target dataset constructed by our research team. The wireless network was set up in a laboratory with a relatively complex environment. The sensors used in the experiment all operate in the 2.4 GHz frequency band, which is the band used by Wi-Fi routers, Bluetooth devices, wireless mice, and keyboards, among other equipment. Additionally, the laboratory has many experimental instruments, tables, and cardboard boxes. The complex experimental environment will lead to an inestimable multipath effect. The parameter configuration of the transmitter (TX) module is as follows: the IQ sampling rate is set to 1 MHz; the carrier frequency is set at 2.4 GHz; the gain is set to 30; the number of samples per symbol is 10,000; and the transmission signal frequency and amplitude are set to 10 kHz and 1.00, respectively. The equipment used for data collection includes an RMC-8357 controller case from NI, a CDA-2990, a PXIe-1095 case, four USRP-2953 devices, and seven antennas.

**Figure 4:**
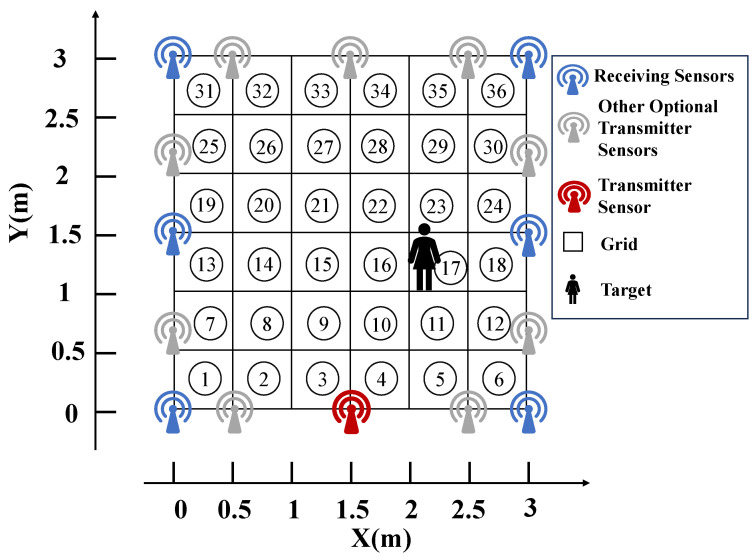
Schematic of the indoor experimental setup.

In our indoor experiment, as shown in Figure 4, the experimental area was set as a 3 m by 3 m square. Six immovable receiving sensors and one movable transmitting sensor, which could move between ten different preset positions, were deployed within this area. The entire wireless network system was mounted on stands 1.3 m above the ground. The subject of the experiment was an individual with a height of 1.66 m.

To ensure the accuracy of the experiment, we divided the area covered by the wireless network into 36 grids of 0.5 m by 0.5 m, with each grid point being considered a valid positioning point. When the target was present at a certain grid position within the network area (a total of 36), the transmitting node emitted radio signals, and all receiving nodes received RSS information, with the transmission and reception process repeated 30 times. Then, the transmitting sensor node changed to the next available transmitting position and emitted radio signals, with all receiving nodes receiving RSS information. All the collected RSS information was assembled into an RSS matrix representing the positional information at the current location. The RSS information collected at each position was divided into two parts: data from 25 trials were used to construct a dictionary, and the remaining 5 trials were used as test signals.

This study extracts signal features based on RSS using the Fourier transform to analyze time-domain signals collected at fixed timestamps. By calculating the amplitude of each harmonic component, the first and second harmonics, which have larger amplitudes, are selected as feature data to enhance the representational capability of the dataset. Therefore, for each position *i* (1≤i≤36), the RSS data training set $Z_{i}\in R^{120\times 25}$, the label set $I_{i}\in R^{1\times 25}$, and the rest are the test set. Finally, the RSS training data of all positions are integrated into a comprehensive dataset $Z\in R^{120\times 900}$, while the test dataset is $p\in R^{120\times 180}$.

According to this working mode, the number of links passing through the entire grid area equals the number of selectable transmitting sensor node positions multiplied by the number of receiving sensor nodes. In the entire sensor network, it is only necessary to deploy (the number of receiving sensor nodes + 1) sensor nodes to provide an adequate number of links. In the case of a very small number of receiving nodes, sufficient wireless link information can be obtained as long as there are enough selectable transmission positions for the sensor nodes. Therefore, achieving high positioning accuracy with a minimal number of sensor nodes is possible [31].

Table 3 presents the values of key parameters for the two proposed models in the indoor experimental environment.

### 4.2. Experimental Evaluation Metrics

When evaluating the performance of the model, we selected the following three quantitative indicators to comprehensively and objectively measure the key performance of the model.

#### 4.2.1. Accuracy

Accuracy is the basic indicator for measuring the localization ability of the model. Assuming $S_{total}$ is the total number of test samples and $S_{correct}$ is the number of accurately positioned samples, the localization accuracy is defined as follows:

(30) $$Accuracy=\frac{S_{correct}}{S_{total}}$$

The position label output by the model is directly compared with the original correct label of the sample. If the two are completely consistent, the sample is determined to be correctly localized and included in the category of $S_{correct}$; if there is a discrepancy, it is judged as a localization error and not included in the statistics of $S_{correct}$. This indicator intuitively reflects the proportion of test samples that the model can correctly locate and is an important basis for evaluating the basic performance of the model.

#### 4.2.2. Average Localization Error (ALE)

This metric is used to quantify the average distance error between the estimated sample position and the target’s true position. Assuming the true position of *N* targets is $\left(x_{1},y_{1}\right),...\left(x_{N},y_{N}\right)$, and the model estimates that the position is $\left(x_{1}^{\prime },y_{1}^{\prime }\right),...\left(x_{N}^{\prime },y_{N}^{\prime }\right)$, then ALE is defined as follows:

(31) $$ALE=\frac{\sum_{i=1}^{N}\sqrt{{\left(x_{i}-x_{i}^{\prime }\right)}^{2}+{\left(y_{i}-y_{i}^{\prime }\right)}^{2}}}{N}$$

By calculating the Euclidean distance between each target position estimate and the true value and taking their average, ALE can accurately reflect the overall performance of the model in localization accuracy and is an important quantitative indicator for measuring the localization accuracy of the model.

#### 4.2.3. Average Computation Time (ACT)

The average computation time is a key indicator for evaluating the efficiency of model operation. It reflects the average time required for the model to process a single test sample. A lower average consumption time means that the model has higher real-time performance and practicality, which is crucial for rapid response in actual application scenarios. The specific calculation method is as follows:

(32) $$ACT=\frac{T_{total}}{S_{total}}$$

where, $T_{total}$ is the total time taken by the model to process all test samples and $S_{total}$ is the total number of test samples.

#### 4.2.4. Cumulative Distribution Function (CDF)

The CDF is used to characterize the cumulative probability distribution property of a random variable. For a random variable *X*, its cumulative distribution function (F(x)) describes the probability of the event that the value of *X* is less than or equal to *x*. It is defined as follows:

(33) F(x)=P(X⩽x)

If the CDF curve at a certain SNR is overall more "left-leaning", then the probability of small errors at this SNR is higher.

#### 4.2.5. Noise Processing

In actual application environments, DFL systems are often disrupted by a variety of external factors, among which noise interference is particularly prevalent and has a significant impact. To fully verify the model’s performance under various noise levels, we deliberately added noise with different signal-to-noise ratios (SNRs) to the test signal. Specifically, let the initial test signal be p, which refers to the RSS used during the testing phase, and the test signal after adding noise be $\tilde{p}$, where S represents Gaussian noise following a Gaussian distribution with different SNRs.

(34) $$\tilde{p}=p+S$$

Here, our initial test signal p, obtained from real-world experimental environments, contains valid positioning information. During propagation, factors such as distance, multipath effects, and other radio frequency interferences reduced its signal strength. As this signal was acquired under actual conditions, it inherently includes signal attenuation and background noise. To evaluate the performance of the proposed method in varying environments, we added Gaussian noise to simulate variable environmental noise. Adding this noise at various signal-to-noise ratio (SNR) levels represents different background noise conditions. This tests the algorithm’s performance under diverse conditions and verifies its stability against various noise disturbances. The purpose of this experimental setup is to test the model’s adaptability and robustness to noisy environments in more realistic, challenging conditions.

SNR is an important parameter that describes the proportional relationship between signal strength and background noise strength. It is a key indicator for measuring the degree of noise interference. In this experiment, we varied the SNR of the test signal from −10 dB to 35 dB in 5 dB increments.

### 4.3. Dimensionality Reduction

In the DFL system, to improve localization accuracy and expand the monitoring area, a large number of nodes are often deployed. Although this can improve the accuracy of localization, it also leads to the high-dimensional characteristics of DFL data. The processing of high-dimensional data is not only computationally complex but also time-consuming. Especially in emergency situations, it is crucial to quickly obtain target location information. Therefore, it is necessary to reduce the total localization time of DFL without significantly sacrificing accuracy. In addition, the DFL algorithm needs to process a large amount of RSS measurement data, which is usually highly correlated. Highly correlated data will cause generalization errors in the model, thereby affecting localization accuracy.

To solve these problems, we use subspace technology [30] to reduce the dimensionality of high-dimensional data. Compared with the traditional independent interpretable algorithm based on the coordinate descent algorithm (CDA), this method can reduce the correlation between features and can significantly reduce the calculation time while ensuring localization accuracy, thereby improving the generalization ability and localization accuracy of the model.

### 4.4. Experimental Results on Outdoor Public Dataset

#### 4.4.1. Performance Evaluation of the Proposed Algorithms on the Outdoor Public Dataset

In this study, we comprehensively compare the two proposed models with several previous state-of-the-art methods based on sparse coding, including sparse coding implemented by the iterative shrinkage threshold algorithm (SC-ISTA) [30], IILasso [41], IIWLasso [42], the independent interpretable proximal operator (IIPO) with the $\ell_{\frac{2}{3}}$ norm [42], FBS-GMC [31], and IIMCP [33].

**Figure 5:**
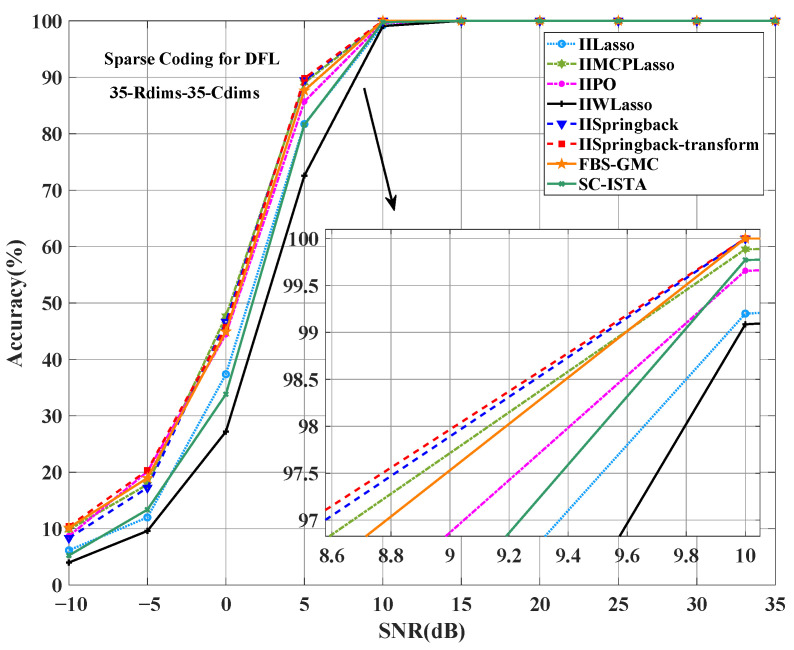
Comparison of accuracy between the proposed two models and other models in the outdoor public dataset.

As can be seen from Figure 5, the accuracy of our two models is better than that of other comparison models at low SNR levels compared to a reference value. Specifically, under the condition of a 5 dB SNR, the accuracy of our IISpringback-transform model reached 89.6%, and the accuracy of the IISpringback model reached 89.43%, both of which are about 1.94 and 1.77 percentage points ahead of the third-ranked IIMCP method. This result shows that our model can show better performance in an environment with higher noise levels.

**Figure 6:**
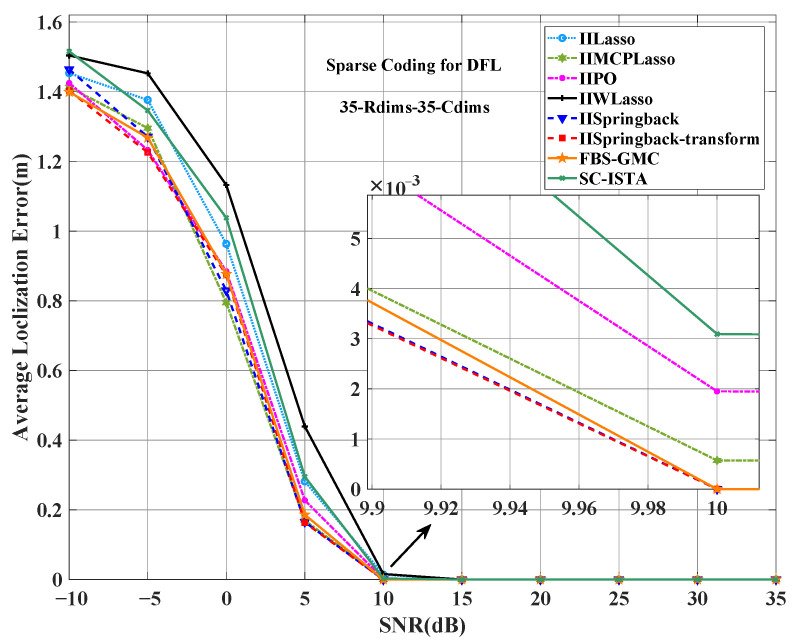
Comparison of ALE between the proposed two models and other models on the outdoor public dataset.

Figure 6 shows the average localization error under different SNRs. In the entire SNR range, the average localization error of our proposed method is the lowest at most SNR levels, and the performance is the best. When the SNR is 10 dB, the average localization error of our method is almost 0, while other methods can only achieve similar low error levels at higher SNRs (such as 15 dB or higher).

#### 4.4.2. Analysis of the CDF of Localization Errors

**Figure 7:**
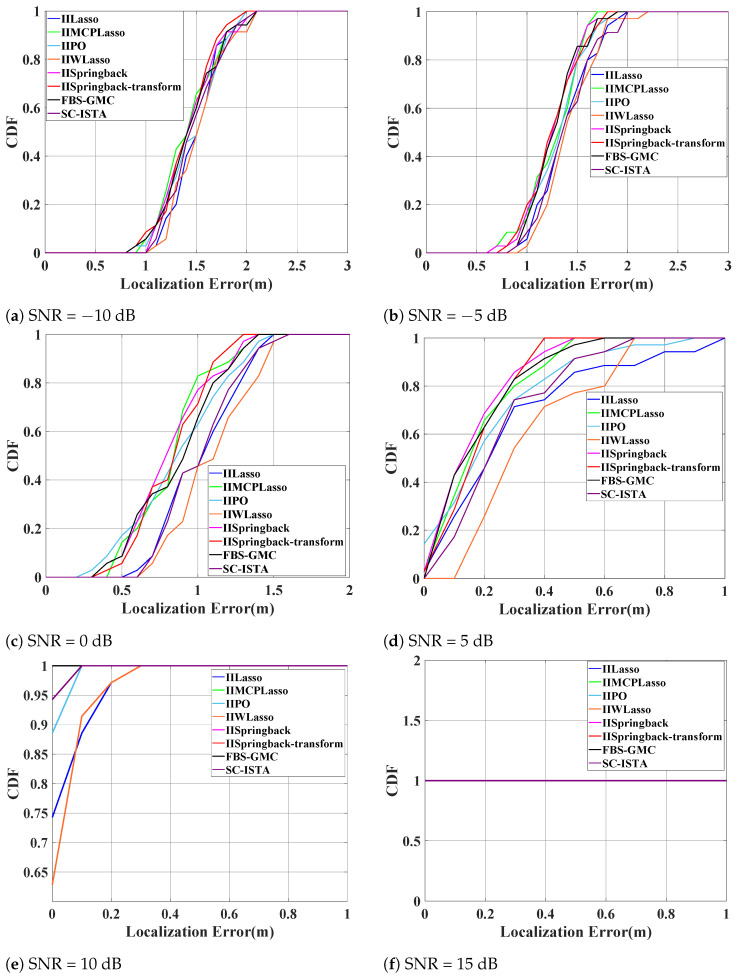
CDF of localization errors at various signal-to-noise ratio (SNR) levels on the outdoor public dataset. Notable overlaps: (**e**) IIMCP and SC-ISTA (for localization errors 0–0.1); (**f**) all eight algorithms’ curves coincide.

Figure 7 consists of six subplots that reflect the distribution of the CDF of localization errors under SNR levels from −10 dB to 15 dB. We can generally observe that as the SNR increases, the cumulative probability curves of localization errors shift to the left, indicating that the positioning accuracy is continuously improving. The CDF value of our proposed model reaches 1 when the localization error is almost the smallest, demonstrating that our proposed model’s performance is superior to other algorithms.

#### 4.4.3. Comparison of Time Efficiency Among Proposed and Existing Algorithms

Next, we tested the execution time of the proposed algorithm and related localization algorithms. Specifically, we used the dimensionality reduction technique mentioned above to reduce the dimension of the outdoor experimental dataset from 784-Rdims-875-Cdims to 35-Rdims-35-Cdims. Through this process, we obtained the results shown in Table 4.

Our method shows significant advantages in terms of time. Specifically, our algorithm only takes $1.01\times 10^{-4}$ and $1.05\times 10^{-4}$ seconds to complete the calculation, which is much lower than other algorithms. Furthermore, the efficient performance of our algorithm when processing dimensionality-reduced data indicates that it has great potential in practical applications. In scenarios that require fast response, such as real-time monitoring or emergency rescue, our algorithm can provide faster localization results, which may save lives or improve operational efficiency.

### 4.5. Indoor Experimental Dataset Results

#### 4.5.1. Performance Evaluation of the Proposed Algorithms on the Indoor Dataset

**Figure 8:**
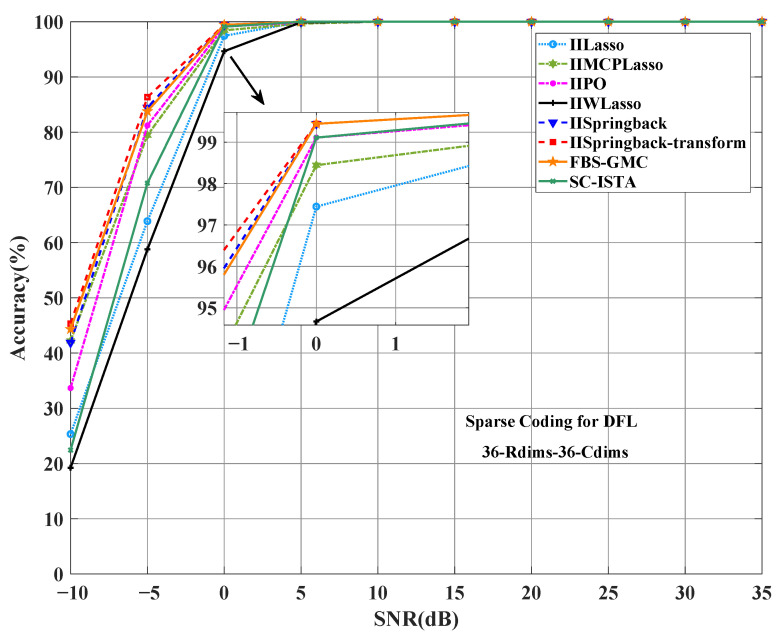
Comparison of accuracy between the proposed two models and other models on the indoor dataset.

**Figure 9:**
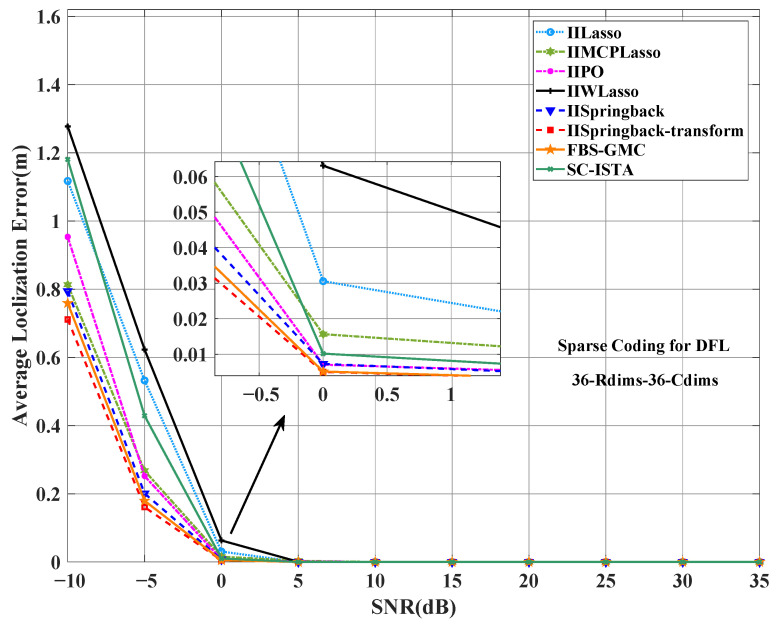
Comparison of ALE between the proposed two models and other models on the indoor dataset.

Similarly, we have conducted a detailed comparison of the proposed model with several existing excellent algorithms on our self-constructed dataset. Figure 8 and Figure 9 show the performance comparison of these algorithms under different SNR conditions. Specifically, our model outperforms other models in terms of accuracy (as shown in Figure 8). Especially in low signal-to-noise ratio environments, such as SNR = −5 dB, our IISpringback-transform model has an accuracy of 86.3%, while the IISpringback model has an accuracy of 84.4%, which are 2.5 and 0.6 percentage points ahead of the third-ranked FBS-GMC algorithm, respectively. This result shows that our model can still maintain a high accuracy under conditions of poor signal quality, showing its potential in practical applications.

Furthermore, Figure 9 shows the performance of different algorithms in terms of average localization error. Under low-SNR conditions, such as from −10 dB to 0 dB, the average localization error of each algorithm is generally large, and the performance difference is more obvious. However, it is worth noting that our model shows a smaller error under these conditions, and the error is significantly lower than that of other models, showing better robustness.

#### 4.5.2. Analysis of the CDF of Localization Errors

**Figure 10:**
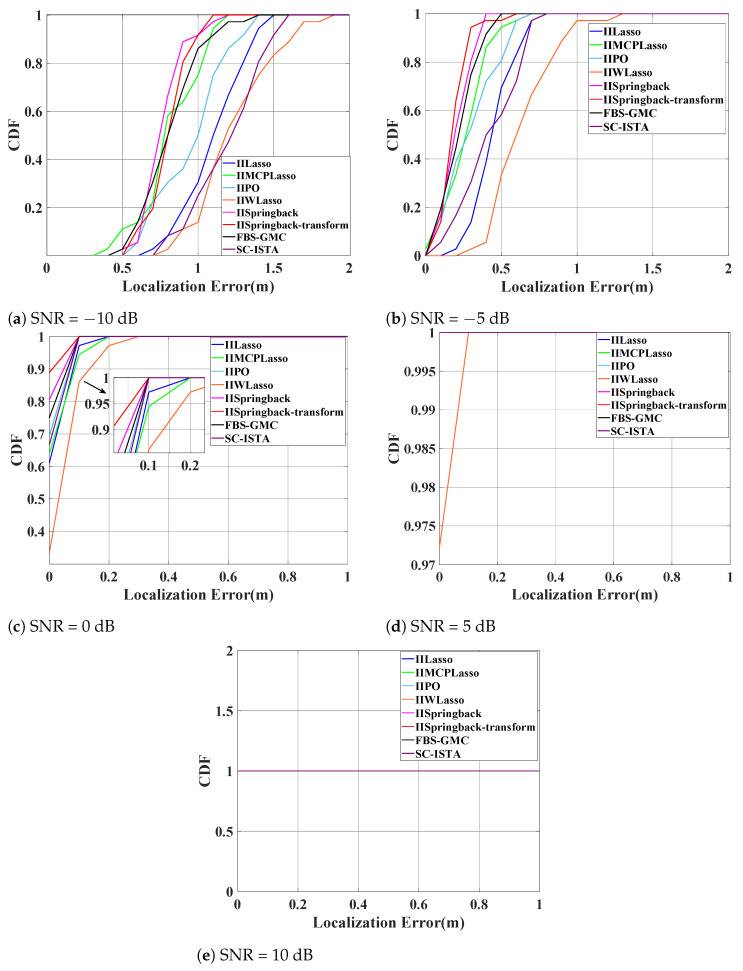
CDF with the localization errors of several SNRs on the indoor dataset (in subplot (**d**), the curves for IIWLasso and IIMCPLasso overlap; in subplot (**e**), the eight curves overlap.).

The five subplots of Figure 10 also illustrate the changes in the CDF with respect to localization errors across a range of SNRs from −10 dB to 10 dB. We can generally observe that the curves of our proposed model are almost all located on the left side of the chart, indicating that when the model reaches higher CDF values, the localization error is minimized. This implies that our model has smaller localization errors at the same cumulative probability level, thereby reflecting higher positioning accuracy under various SNR conditions. Furthermore, the positions of these curves also demonstrate the superior performance of our model compared to other algorithms under various noise levels, further confirming the robustness and effectiveness of our model.

#### 4.5.3. Comparison of Time Efficiency Among Proposed and Existing Algorithms

We tested the time consumption of different DFL algorithms on the original dataset of 120-Rdims-900-Cdims (without dimensionality reduction). The results are shown in Table 5.

Our method demonstrates optimal time efficiency in processing higher-dimensional data, enabling our algorithm to support real-time localization. This is crucial for applications requiring instant feedback, such as navigation, virtual reality, and military target localization.

### 4.6. Ablation Experiment

This section aims to evaluate the key parameters of the IISpringback model and its variant, IISpringback-transform. The experiments focus on two main aspects: the effectiveness of transform learning and the impact of the inter-column coherence of the dictionary ($B_{2}\left(x\right)$ sparsity constraint) on model performance.

To ensure the objectivity and comparability of the experimental results, all ablation experiments are performed on a public outdoor dataset and in an experimental environment. By systematically adjusting and comparing the model performance under different configurations, we can clearly identify the specific contributions of transform learning and the coherence constraints to model performance.

#### 4.6.1. The Impact of Transform Learning on Model Performance

**Figure 11:**
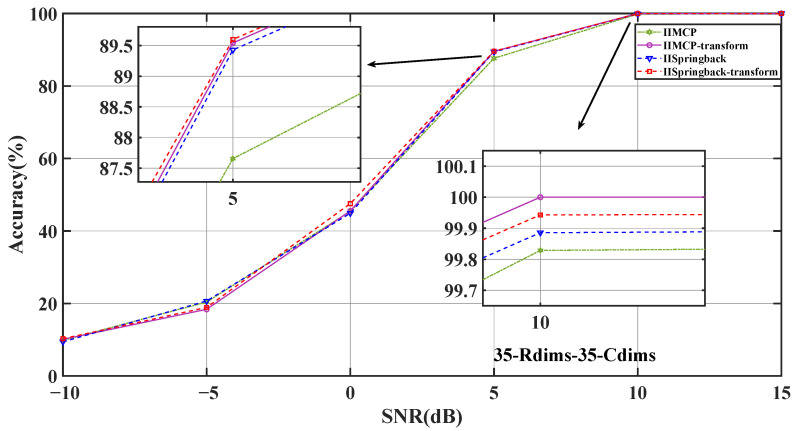
The impact of transform structure on the accuracy of models.

In this study, we further investigate the impact of transform learning on model performance. To this end, we have improved the IIMCP algorithm and introduced the IIMCP-transform algorithm based on transform learning. We compared this new algorithm with the two models we proposed, and the experimental results are shown in Figure 11 and in Table 6.

The experimental results indicate that at an SNR of −5 dB, the synthetic model’s accuracy is slightly higher than that of the model employing transform learning. However, this level of accuracy remains relatively low and lacks practical significance. At all other SNR levels, the model utilizing transform learning demonstrates superior accuracy, significantly outperforming the synthetic model.

We then conducted a time consumption test on the outdoor dataset that had undergone dimensionality reduction (input dimension was 35 × 35). The test results are shown in Table 7.

Based on the experimental outcomes, our analysis-based IIMCP demonstrates superior performance over the synthetic IIMCP algorithm in terms of accuracy and computational cost. Similarly, the analysis-based Springback outperforms the synthetic Springback algorithm in these aspects. These improvements are primarily attributed to the application of the analysis-based transform learning approach. This method directly learns analytical transformations from signal data, enabling the extraction of sparse features. During this process, the overall structure of the signals is effectively preserved, and the method exhibits greater efficiency when processing large-scale datasets, thereby enhancing the performance of the algorithms.

The analysis-based transform learning approach provides a more direct and efficient means of feature extraction compared to synthetic methods. By learning transformations that are tailored to the characteristics of the signal data, this approach not only maintains the integrity of the signal structure but also optimizes the computational process. This is particularly beneficial when dealing with large volumes of data, as it reduces the time required for processing without compromising on accuracy. Consequently, the algorithms that incorporate this approach achieve better overall performance, making them more suitable for applications that demand both speed and precision.

#### 4.6.2. The Influence of $B_{2}\left(x\right)$ Coherence Constraints on Model Performance

**Figure 12:**
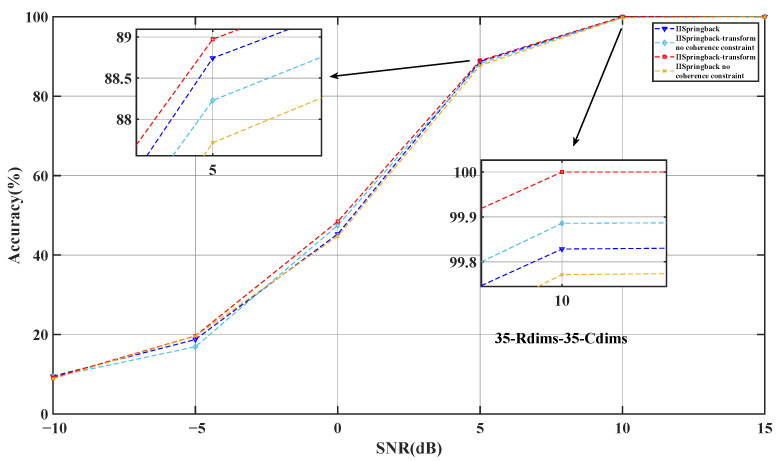
The impact of coherence constraints on the accuracy of IISpringback and IISpringback-transform models.

In order to verify the specific impact of the coherence constraint on the performance of the proposed IISpringback model and IISpringback-transform model, we designed ablation experiments. These experiments are designed to evaluate the performance of the model without considering the coherence constraint and compare it with the performance after introducing the constraint, as shown in Figure 12 and in Table 8. Through this comparative analysis, we can more accurately understand the role of the coherence constraint in improving the performance of the model.

Experimental results show that the introduction of coherence constraints significantly improves the accuracy and robustness of the model when dealing with highly correlated data. In addition, the introduction of these constraints also makes the decision-making process of the model more transparent, thereby improving the interpretability of the model. These findings further confirm the importance of coherence in designing efficient and interpretable sparse recovery models.

The results of two ablation experiments indicate that introducing transform learning and optimizing the coherence between dictionary columns both significantly enhance the model’s accuracy and reduce computational time. These achievements not only validate the effectiveness of our model design but also provide valuable references for future applications in similar tasks.

### 4.7. Discussion

#### 4.7.1. Analysis of Experimental Results

Based on the experimental results, this paper discusses regularization, performance, transform learning, and complexity analysis.

**1.** The DFL method proposed in this study, based on the Springback penalty function, significantly enhances positioning accuracy in complex environments by achieving a dynamic balance between sparsity and computational complexity. Experimental data indicate that, compared to conventional non-convex regularization techniques, the Springback penalty function is more efficient in capturing the sparsity characteristics of signals and effectively avoids the issues of overfitting and underfitting.

**2.** The experimental results demonstrate that, compared to existing advanced sparse coding models, the two models proposed in this paper (IISpringback and IISpringback-transform) show significant advantages in low-SNR environments: (a) Higher positioning accuracy: In indoor environments with a low SNR, such as SNR = −5 dB, the IISpringback-transform model achieved an accuracy of 84.94%, and the IISpringback model reached 84.28%, outperforming the third-ranked IIPO algorithm by 5.32 and 4.66 percentage points, respectively. This indicates that our models can maintain high accuracy even under poor signal conditions, showing their potential for practical applications. (b) Higher computational efficiency: Whether processing reduced-dimensionality data or non-reduced data, the processing time of our algorithm is significantly shorter than other algorithms, achieving optimal time efficiency. This is crucial for real-world scenarios requiring rapid response, such as real-time monitoring or emergency rescue.

**3.** Compared to the standalone Springback model, the Springback-transform model, which employs transform learning, achieves better positioning performance while reducing computational overhead. The reasons are as follows: (a) Analytical transform learning directly learns the analytical transform from signal data to extract higher-quality sparse features, enabling sparse representation of signals in the transform domain. This sparsity can more effectively capture the key features of signals and eliminate redundant information. (b) This analytical framework is more efficient when processing large-scale data because it avoids the complex matrix decomposition calculations required for each signal sample in synthetic models. This leads to dual improvements in positioning accuracy and computational efficiency.

**4.** We have conducted a Big O analysis of the proposed IISpringback and IISpringback-transform models compared to the IIMCP algorithm. Assuming the signal dimension $X\in R^{k\times n}$ and the dictionary dimension $D\in R^{k\times m}$, with T representing the number of iterations, the complexity for all of them is $O(T\times \left(m^{3}+m^{2}n+mkn\right))$, where *m*, *n*, and *k* are the dimensions of the signal, dictionary, and the number of iterations, respectively.

#### 4.7.2. Limitation Analysis and Future Prospects

**1.** Dataset Limitations: The current experiments only utilized a public dataset and a laboratory indoor dataset. The outdoor data was deployed on a grassy area near the Merrill Engineering Building at the University of Utah, which is a relatively ideal environment and does not verify the model’s generalization capability in more complex settings such as multi-story buildings and high-density obstructions. The indoor sensor deployment was limited to a 3m×3m area, which may not fully reflect the model’s performance across various indoor scenarios, thus affecting a comprehensive evaluation of the model. Future work will introduce testing in complex scenarios, expand the indoor test area to 10m×10m, and simulate real-life layouts like homes and hospitals.

**2.** Insufficient Adaptability to Dynamic Environments: The RSS data collection involved only stationary targets within a grid, without considering moving targets or multi-target interactions. Dynamic interferences, such as people walking and object occlusions, could significantly impact performance in practical applications. Future research will extend from single-target static positioning to multi-target dynamic scenarios, design experiments with moving targets to record dynamic RSS changes, and investigate signal compensation mechanisms under occlusion conditions.

**3.** Limitations in Noise Handling: The experiments only tested Gaussian noise with an SNR ranging from −10 dB to 35 dB, whereas real environments may encounter non-Gaussian interferences like impulse noise and multipath effects. Future work will involve constructing a composite noise test set that includes impulse noise (Poisson distribution), multipath effects, and inter-device interference, introducing a robust signal processing module, and establishing a more comprehensive set of noise robustness evaluation indicators.

## 5. Conclusions

This paper introduces an innovative sparse coding method based on the Springback weakly convex penalty function and further develops the IISpringback-transform model, which is grounded in a transform learning framework. The essence of our approach is the introduction of a novel weakly convex penalty function known as the Springback penalty. This function artfully integrates a sparsity-promoting compression component with a signal amplitude-preserving rebound component, dynamically balancing sparsity and computational complexity via the parameter θ. This design significantly enhances the accuracy and computational efficiency of positioning in complex environments.

Looking to the future, we intend to extend the models and algorithms presented in this paper to more sophisticated positioning scenarios, such as multi-target localization and dynamic environment localization. Additionally, we will continue to explore the potential integration of our proposed models with other cutting-edge technologies to further refine model performance, enhance positioning accuracy and efficiency, and thereby advance the field of device-free localization technology.

## Figures and Tables

**Table 1:** Notations.

Notations	Description
*T*,*R*	The number of transmitting sensors *T* and receiving sensors *R*
R	The sets of real numbers
$R^{R\times T}$	R×T matrices with real numbers
$\alpha_{i,j}$	The RSS value received by the *i*-th receiving sensor from the *j*-th transmitting sensor, where i∈{1,2,…,R} and j∈{1,2,…,T}
$\alpha_{i}$	A vector composed of RSS measurements of the *i*-th receiving sensor node
γ	RSS measurement matrix
$\gamma_{n,b}$	The *b*th RSS matrix measured at the *n*th RP
$w_{nb}$	The vector of samples defined in Equation (2)
$W_{n}$	The sample matrix of the *n*-th RP is defined in Equation (3)
W	The dictionary, as defined in Equation (4)
y	The observation signal
$x_{cj}$	The sparse coefficient of $w_{cj}$, as defined in Equation (5)
λ, β	Regularization parameters, as defined in Equation (6)
θ	Model parameters for the Springback penalty, as defined in Equation (7)
G	The symmetric matrix in the correlation constraint among dictionary columns, as defined in Equation (8)
$G_{\mathrm{ij}}$	The coherence between dictionary columns $G_{i}$ and $G_{j}$, as defined in Equation (9)
$x^{*}$	The sparse vector obtained by transforming *x*, which is obtained by summing $x_{pj}$, as defined in Equations (10) and (11)
*s*	The index of a specific element in the sparse vector $x^{*}$, which is also the number of the estimated test position, is defined in (12)
β	Regularization parameter for the correlation constraint, as defined in Equation (13)
D	Transform matrix (dictionary)
Prox	The soft threshold operator
π	Step size, as defined in Equation (29)
S	Gaussian noise following a Gaussian distribution, as defined in Equation (34)

**Table 2:** Key parameters of the proposed model in public outdoor experimental environment.

Model	θ	λ	β
IISpringback	0.7	$5\times 10^{-2}$	$1\times 10^{-4}$
IISpringback-transform	0.7	$5\times 10^{-2}$	$1\times 10^{-5}$

**Table 3:** Key parameters of the proposed model in indoor experimental environment.

Model	θ	λ	β
IISpringback	0.7	$5\times 10^{-2}$	$1\times 10^{-5}$
IISpringback-transform	0.7	$5\times 10^{-2}$	$1\times 10^{-4}$

**Table 4:** The time cost of different DFL algorithms when the input data dimension is 35-Rdims-35-Cdims.

Algorithms	Time(s)
SC-ISTA	$4.72\times 10^{-4}$
IILasso	$3.39\times 10^{-3}$
IIWLasso	$1.08\times 10^{-2}$
IIPO	$3.97\times 10^{-3}$
FBS-GMC	$3.43\times 10^{-4}$
IIMCP	$1.19\times 10^{-4}$
IISpringback(Our)	$1.05\times 10^{-4}$
IISpringback-transform(Our)	** $1.01\times 10^{-4}$ **

**Table 5:** The time cost of different DFL algorithms when the input data dimension is 120-Rdims-900-Cdims.

Algorithms	Time(s)
SC-ISTA	0.166
IILasso	0.498
IIWLasso	1.522
IIPO	0.071
FBS-GMC	0.260
IIMCP	0.051
IISpringback(Our)	0.049
IISpringback-transform(Our)	**0.047**

**Table 6:** Comparison of model accuracy affected by transform learning under different SNR levels.

Framework	Algorithms	Category	SNR(dB)			
			−5	0	5	10
Transform learning	IIMCP	Analytical	18.40%	**45.54%**	**89.54%**	**100%**
		Synthetic	**20.4%**	45.43%	87.66%	99.83%
	Springback	Analytical	18.91%	**47.54%**	**89.60%**	**99.94%**
		Synthetic	**20.69%**	44.91%	89.43%	99.89%

**Table 7:** Impact of transform learning on model time consumption with input data dimensions of 35-Rdims-35-Cdims.

Algorithms	Time(s)
IIMCP	$1.19\times 10^{-4}$
IIMCP-transform(Our)	$1.15\times 10^{-4}$
IISpringback(Our)	$1.05\times 10^{-4}$
IISpringback-transform(Our)	$1.01\times 10^{-4}$

**Table 8:** Comparison of model accuracy affected by coherence constraints under different SNR levels.

Framework	Algorithms	Whether It Has	SNR(dB)			
			−5	0	5	10
$B_{2}\left(x\right)$ Coherence Constraints	IISpringback	Yes	18.74%	**45.31%**	**88.74%**	**99.83%**
		No	**19.6%**	44.74%	87.71%	99.77%
	IISpringback-transform	Yes	**19.66%**	**48.46%**	**88.97%**	**100%**
		No	16.91%	47.49%	88.23%	99.89%

## Data Availability

The raw data supporting the conclusions of this article will be made available by the authors on request.
